# The effect of postmortem time on the RNA quality of human ocular tissues

**Published:** 2013-06-11

**Authors:** Byung-Jin Kim, Nicholas Sprehe, Ashley Morganti, Robert J. Wordinger, Abbot F. Clark

**Affiliations:** 1 Department of Cell Biology & Anatomy and The North Texas Eye Research Institute, University of North Texas Health Science Center, Fort Worth, TX; 2 Lions Eye Institute for Transplant and Research, Tampa, FL

## Abstract

**Purpose:**

Profiling gene expression in human ocular tissues provides invaluable information for understanding ocular biology and investigating numerous ocular diseases. Accurate measurement of gene expression requires high-quality RNA, which often is a challenge with postmortem ocular tissues.

**Methods:**

We examined the effect of various death to preservation (DP) times on the RNA quality of ten different ocular tissues. We used 16 eyes from eight different human donors. The eyes were preserved immediately in RNA*later* or preserved after initial storage at 4 °C to create a range of DP times from 2 to 48 h. Ten ocular tissues were dissected from each eye. After total RNA was extracted from each dissected ocular tissue, the RNA integrity number (RIN) was determined using an Agilent Bioanalyzer.

**Results:**

The RIN values from corneal and trabecular meshwork tissues were significantly (p<0.05) higher than those from the ciliary body at an earlier DP time (<6 h), but were not different among all tissues after 8 h. Interestingly, the RIN values from non-vascularized tissues were significantly (p=0.0002) higher than those from vascularized ocular tissues at early DP times (<6 h). The RIN value from the cornea was significantly (p<0.05) higher at short DP times compared to longer DP times. The RIN values from corneal tissues were significantly correlated to DP time according to regression analysis (p<0.05).

**Conclusions:**

In this study, we determined RNA quality from postmortem ocular tissues with various DP times. Our results emphasize the need for rapid preservation and processing of postmortem human donor eye tissues, especially for vascularized ocular tissues.

## Introduction

Messenger ribonucleic acid (mRNA) mediates informational transfer from genomic DNA to biologic effector protein molecules. Gene expression profiling using RNA isolated from cells and tissues has been extensively used to study the development, differentiation, and effects of exogenous factors such as growth factors and drugs, as well as to better understand disease pathogenesis. Although isolating RNA from fresh cell cultures is relatively straightforward, harvesting RNA from tissues, particularly human tissues, is more challenging. RNA integrity in tissues depends on the postmortem time to preservation, the metabolic profiles of the tissues, endogenous RNase activity, and natural RNA degradation.

Numerous studies have been performed to study mRNA expression profiles using various techniques, including reverse transcription–polymerase chain reaction (RT–PCR), RNA differential display, DNA microarrays, and RNAseq. In particular, microarray analysis of mRNA expression has been used to identify genes associated with aging [1], retinal stem/progenitor cell biology [2], as well as the pathophysiology of certain diseases such as age-related macular disease (AMD) [3], rod-cone dystrophy [4], retinitis pigmentosa (RP) [5], and glaucoma [6]. DNA microarrays have been used to profile genome-wide gene expression in multiple human ocular tissues to investigate the physiology and pathophysiology of the eye [7]. However, several obstacles exist to obtaining high-quality RNA from human donor eyes.

RNA is progressively degraded in postmortem tissues and during RNA extraction. In many cases, RNA extracted from donor eye tissues is not of optimal quality, which complicates gene expression analysis. Several methods are used to determine RNA quality. A commonly used technique is reverse transcriptase PCR amplification of housekeeping genes such as β-actin or *GAPDH*. RNA integrity is also tested by visualizing 28S and 18S rRNA after agarose gel electrophoresis. This method is now automated using a microcapillary electrophoresis system to provide an RNA integrity number (RIN) [8]. There is a spectrum of RIN numbers from the lowest integrity (1=total degradation) to the highest integrity (10=intact) [8]. This technique was compared to the traditional RT–PCR-based RNA integrity test, and there was good correlation between these two techniques [8,9].

Several studies have been performed to establish optimal conditions for RNA extraction from human ocular tissues. Wang and colleagues tested several different RNA preservation methods for human donor ocular tissues to extract high-quality RNA [10]. They found that death to preservation (DP) time, preservation in RNA*later*, and the RNA extraction technique all affected overall RNA quality. Malik and colleagues examined the effects of DP time on RNA quality in the retina and retinal pigment epithelium of pig eyes [11]. Immediate postmortem enucleation as well as rapid dissection and freezing provided the best mRNA quality.

Although many of the eye tissues are vascularized, the eye also contains avascular tissues (i.e., the cornea, lens, trabecular meshwork, and sclera). DP time may differentially affect RNA quality in vascularized versus non-vascularized tissues. In the current study, we examined RNA quality in ten different vascularized and avascular human ocular tissues at various postmortem times using RIN analysis.

## Methods

### Human donor eyes and preservation

Donor eyes were provided by the Lion’s Eye Institute for Transplant and Research (LEITR; Tampa, FL). As shown in Table 1, 16 eyes from eight donors were collected at different DP times before preservation in RNA*later* (Ambion Inc., Austin, TX) as described earlier [10]. Paired eyes were received and processed at LEITR within 2–6 h of death. A 2̴̴̴ 3 cm incision was made through the sclera and retina at the equator of each eye. One eye was immediately placed in 30 ml of RNA*later* and stored at 4 °C. The contralateral eye was placed in a moist chamber at 4 °C for 8–48 h before being placed in 30 ml of RNA*later*. The paired eyes were then shipped on ice to UNTHSC for dissection.

**Table 1:** Human donor eyes used in study.

ID				RIN									
EYE#	M/F	OD/OS	DP(h)	ON	I	CB	C	R	C/R	S	ONH	TM	L
1	M	OD	48	4.7	6.0	5.6	2.4	6.9	6.2	5.9	5.6	8.5	
2		OS	04	2.7	5.1	2.4	2.8	4.6	4.1	5.7	1.1	6.1	
3	M	OD	08	4.9	6.2	5.3	7.3	6.9	3.4	5.2	7.0	5.4	
4		OS	05	4.4	6.7	2.3	8.5	2.4	5.2	6.0	7.8	9.0	
5	F	OD	24	2.0	3.8	7.0	3.2	6.3	2.9	5.8	4.7	3.0	
6		OS	02	5.2	6.7	7.3	6.2	3.5	4.7	5.4	7.1	7.5	
7	M	OD	10	3.7	2.9	7.3	4.0	4.8	2.4	5.5	6.1	4.6	
8		OS	06	5.5	5.5	2.5	7.6	4.0	3.6	2.9	6.2	7.6	
9	F	OD	04	6.8	5.1	5.5	9.0	2.2	5.4	5.7	8.4	3.4	6.8
10		OS	19	5.5	3.7	3.5	6.7	7.3	4.9	7.0	7.0	1.9	5.8
11	M	OD	04	7.1	6.1	5.8	8.6	3.1	5.0	6.8	7.5	8.2	
12		OS	12	7.0	5.0	4.7	7.5	6.0	4.1	3.0	7.3	7.1	
13	M	OD	05	7.0	6.0	2.4	7.4	7.5	6.4	7.5	7.6	8.7	6.7
14		OS	16	6.6	7.8	2.2	8.2	6.2	5.3	6.0	8.3	8.9	7.2
15	F	OD	05	6.7	2.5	2.1	6.5	6.9	6.3	6.1	8.4	8.6	
16		OS	12	2.4	5.4	5.0	2.4	2.4	2.2	1.9	1.0	2.4	

The profiles (sex, OS/OD, DP times, and RIN values) of human eyes are listed. The donor eyes were provided by LEITR. Abbreviations for tissues, ON: optic nerve, I: iris, CB: ciliary body, C: cornea, R: retina, C/R: choroid/retinal pigment epithelium, S: sclera, ONH: optic nerve head, TM: trabecular meshwork, L: lens

### Donor eye dissection

The following ocular tissues were carefully dissected from each donor eye: iris (I), sclera (S), trabecular meshwork (TM), cornea (C), retina (R), optic nerve head (ONH), optic nerve (ON), lens (L), choroid/retinal pigment epithelium (C/R), and ciliary body (CB). The majority of the eyes were pseudophakic, and the crystalline lens was present in only four donor eyes. In some eyes, clean tissue dissection was difficult, especially in eyes that had not been placed in RNA*later* until >12 h DP time. Each dissected tissue was then placed in a 1.5 ml tube filled with RNA*later* and stored in −80 °C until RNA extraction was performed.

### RNA extraction

Each eye tissue was homogenized in 1 ml of Iso-RNA Lysis Reagent (5 PRIME, Gaithersburg, MD) using a TissueLyser II (Qiagen, Germantown, MD). Total RNA was further extracted using RNeasy Micro Kits (Qiagen) as described in the manufacturer’s guide. Briefly, 50 μl of 4-Bromoanisole (BAN; Molecular Research Center, Cincinnati, OH) was added to tissue homogenates and mixed gently. After centrifugation at 12,000 ×g for 15 min at 4 °C, the aqueous phase was separated and mixed with the same volume of 100% ethanol. The aqueous phase and ethanol mixture was passed through an affinity column for RNA and washed. The column was washed again after DNase treatment and dried to remove the remaining ethanol. Total RNA was eluted in DNase/RNase free water.

### Determination of RNA integrity number

The RNA integrity number (RIN) was determined in ten ocular tissues from 16 donor eyes using an Agilent 2100 Bioanalyzer (Agilent Technologies, Santa Clara, CA) with RNA nano- or picochip kit systems depending on the RNA concentration of each ocular tissue (Table 1). First, RNA nano- or picochips were primed using the chip priming station as described in the manufacturer’s instructions. The marker solution was loaded into each well of the RNA nano- or picochips followed by the loading of size markers and RNA samples. The chips were placed in an Agilent 2100 Bioanalyzer, and the RINs were determined using 2100 Expert Software (Agilent Technologies).

### Statistical analysis

Statistical analysis was performed using GraphPad Prism Version 5.0 (GraphPad Software, San Diego, CA). For determining the time-dependent changes in the RIN, linear regression was performed to determine the R^2^ value and the significance between the DP time and the RIN. Data were also subjected to the paired Student *t* test or one-way analysis of variance (ANOVA) followed by a post hoc test (Bonferroni) for differences in the RINs between tissues. All data are expressed as means ± standard error of the mean (SEM). A p value of 0.05 or less was considered statistically significant.

## Results

**Figure 1:**
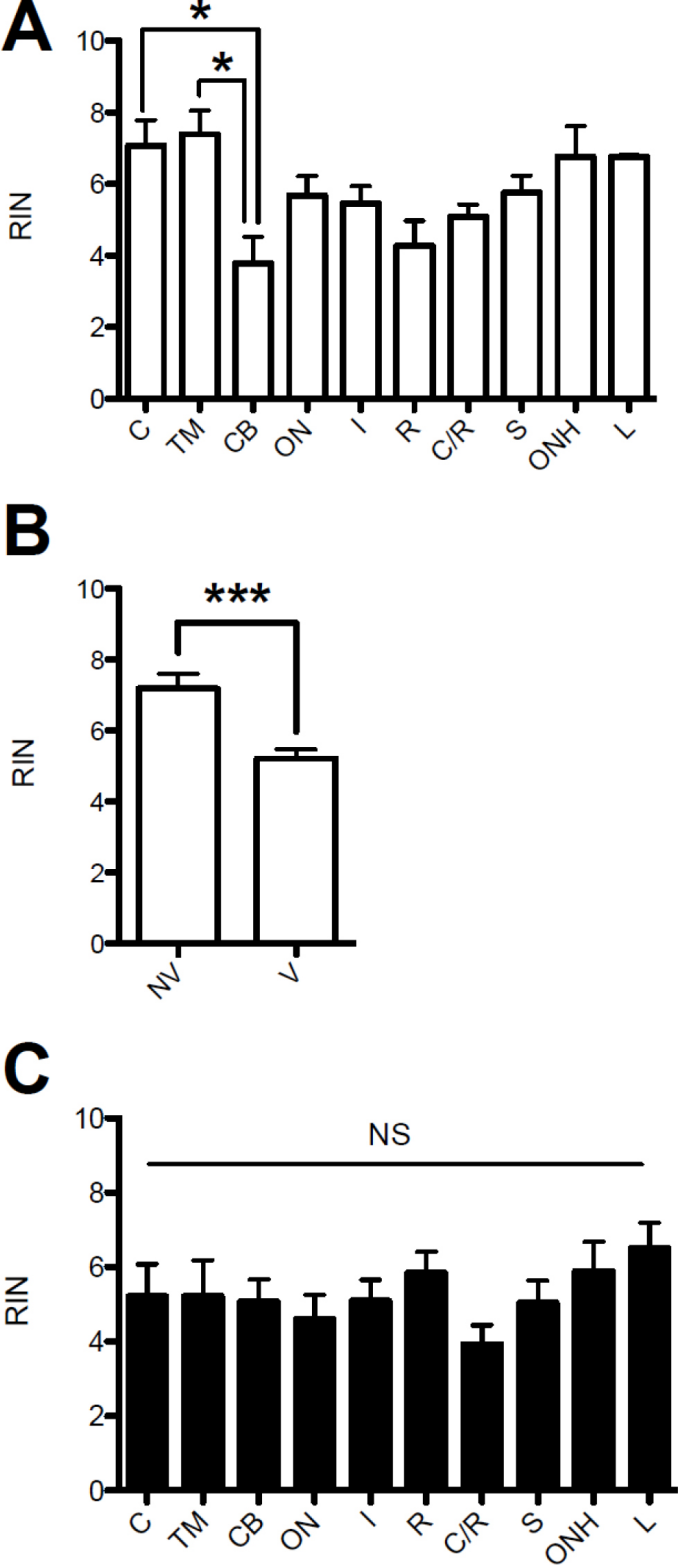
Ribonucleic acid integrity number values from ten ocular tissues with postmortem times <6 and >8 h. **A**: RNA integrity number (RIN) values of ocular tissues that were preserved in RNA*later* within 6 h postmortem. The RIN values of the cornea and TM tissues were significantly higher than the RIN value of the ciliary body (p<0.05). **B**: RIN values for RNA isolated within 6 h. postmortem in non-vascularized (NV) ocular tissues and vascularized (V) ocular tissues. RIN values were significantly higher for NV compared to V tissues (p=0.0002). **C**: RIN values for RNA isolated ocular tissues >8 h. postmortem. There were no significant differences in RIN values among the different ocular tissues. Abbreviations for tissues, C: cornea, TM: trabecular meshwork, CB: ciliary body, ON: optic nerve, I: iris, R: retina, C/R: choroid/retinal pigment epithelium, S: sclera, ONH: optic nerve head, L: lens.

RIN values were determined in ten ocular tissues from 16 donor eyes with various DP times (Table 1). RNA quality varied between ocular tissues processed at <6 h of DP time. The average RIN values varied from 4 to 7 among the ocular tissues placed in RNA*later* within 6 h. of DP. RNA isolated from the cornea and the TM had significantly higher RIN values than RNA isolated from the ciliary body (Figure 1A; p<0.05). The overall RNA quality was significantly higher in non-vascular (NV) tissues (i.e., the cornea, lens, TM, and sclera) compared to the remaining vascularized (V) tissues (Figure 1B; p=0.0002). However, when RNA was extracted from tissues with DP times >8 h, the RIN values for all tissues were <6, and there were no statistically significant differences in RIN values between tissues (Figure 1C).

**Figure 2:**
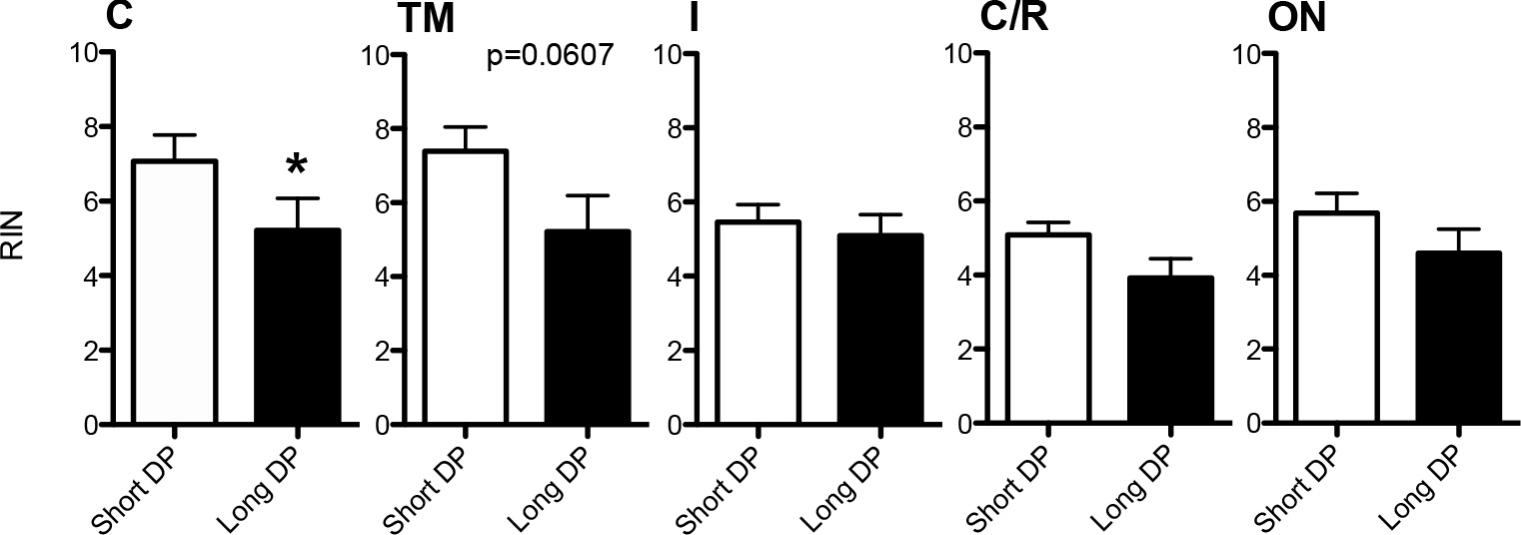
Effect of short versus long postmortem times on RNA quality in five ocular tissues. RNA integrity number (RIN) values for RNA isolated from donor tissues at death to preservation (DP) time ranges (short DP time <6hr or long DP time >8 h; n=8 for each tissue). RIN values were higher with short DP times in the cornea (p<0.05) and TM (p=0.0607). There was no significant difference in RIN values between short and long DP times for the other tissues. Abbreviations for tissues; C: cornea, TM: trabecular meshwork, ON: optic nerve, I: iris, C/R: choroid/retinal pigment epithelium.

**Figure 3:**
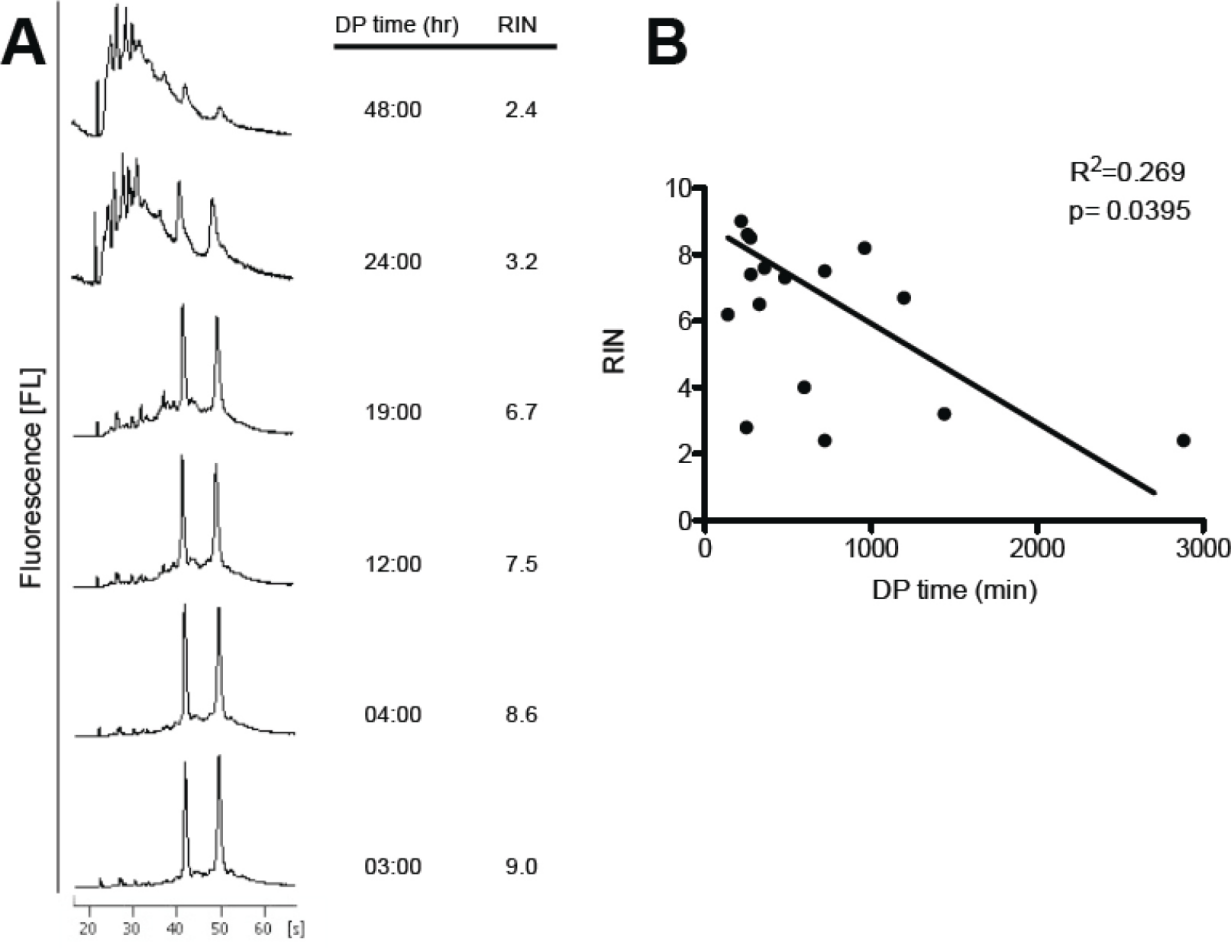
Correlation between death to preservation time and RNA quality in the cornea. **A**: Capillary electropherograms of corneal RNA and corresponding RNA integrity number (RIN) values at varying death to preservation (DP) times. **B**: Correlation between DP time and corneal RIN values assessed with linear regression analysis; p=0.0395; R^2^=0.269 (n=16).

Shorter DP times enhanced RIN values in RNA isolated from the cornea (p<0.05), TM (p=0.0607), and ON, but had only modest to no effects on RNA quality from the iris and choroid/RPE (Figure 2). Representative RIN histograms for corneal RNA are shown based on different postmortem times (Figure 3A). In regression analysis, the RIN from corneal RNA was significantly (p<0.05) correlated with the DP time (Figure 3B; R^2^=0.26903). In contrast, the other ocular tissues did not show significant correlations between the RIN and the DP time.

## Discussion

We examined the effect of postmortem time on the quality of RNA isolated from human ocular tissues subsequent to RNA*later* preservation. Ocular tissue RNA quality was determined as RIN values using a commercial bioanalyzer. We found that RNA quality deteriorated rapidly and was associated with DP time. There was a statistically significant correlation in RIN values with DP times in corneal tissues, but surprisingly not in the other ocular tissues. RNA quality was significantly higher at shorter DP times for the non-vascularized ocular tissues compared to the vascularized ocular tissues.

The quality of the postmortem human DNA was previously determined from the retina and iris. Wang et al. showed that total DNA from the postmortem human retina was significantly superior in quality and quantity compared to that from the postmortem human iris [12]. More relevantly, Malik and colleagues also simulated human donor eye postmortem times using porcine ocular tissues [11]. They studied RNA stability from RPE cells and retina tissues through RT–PCR analysis of housekeeping mRNAs and microchip analysis of 28S and 18S rRNA at various postmortem times. In the retina, expression of the abundant photoreceptor gene *Rho* was detected at all postmortem time ranges out to 48 h. However, in isolated RPE cells, the RPE-specific *RPE65* mRNA showed relatively rapid decay after death. Their results demonstrated that RNA degradation varied by tissue and by the specific RNA analyzed. We also examined the effects of postmortem time on RNA integrity using ten different ocular tissues. One of the paired eyes was placed in RNA*later* within 2–6 h, while the contralateral eye was placed in a moist chamber at 4 °C, simulating normal storage conditions for enucleated human eyes. These eyes were then placed in RNA*later* 8–48 h postmortem (Table 1). We were somewhat surprised to find a good correlation between RNA quality and postmortem time for the corneal tissues only (Figures 3A,B). However, there was a significant correlation between the RNA quality and tissue vascularization with time to preservation <6 h (Figure 1B and Figure 2). RNA from avascular tissues had significantly higher RIN values compared to RNA isolated from vascularized tissues (Figure 1B). We speculate that the metabolic demands of vascularized tissues are more sensitive to the loss of nutrients postmortem, which leads to greater tissue and RNA degradation. It is also possible that the intrinsic RNase activity is higher in vascularized tissues. However, RNA quality was equally compromised in the majority of ocular tissues after 8 h postmortem (Figure 1C).

In this study, we determined the RNA integrity of ten human ocular tissues isolated from human donor eyes preserved at various postmortem times. From our data, we conclude that donor eyes should be preserved in RNA*later* as soon as possible after enucleation to prevent potential loss of RNA quality. Importantly, an incision should be made through the sclera into the vitreous to facilitate penetration of this reagent into the eyes, and eyes should be kept at 4 °C until dissected, preferably no longer than 1 month. RNA quality diminished quickly, particularly in highly vascularized ocular tissues. Our findings provide guidance for analyzing gene expression from human donor ocular tissues.

## References

[1] Yoshida S, Yashar BM, Hiriyanna S, Swaroop A (2002). Microarray analysis of gene expression in the aging human retina.. Invest Ophthalmol Vis Sci.

[2] Jasty S Srinivasan P Pasricha G Chatterjee N Subramanian K Gene expression profiles and retinal potential of Stem/Progenitor cells derived from human iris and ciliary pigment epithelium. Stem Cell Rev 20128:1163-77 22744312 10.1007/s12015-012-9394-3

[3] Kurji KH, Cui JZ, Lin T, Harriman D, Prasad SS, Kojic L, Matsubara JA (2010). Microarray analysis identifies changes in inflammatory gene expression in response to amyloid-beta stimulation of cultured human retinal pigment epithelial cells.. Invest Ophthalmol Vis Sci.

[4] Yzer S, van den Born LI, Zonneveld MN, Lopez I, Ayyagari R, Teye-Botchway L, Mota-Vieira L, Cremers FP, Koenekoop RK (2007). Molecular and phenotypic analysis of a family with autosomal recessive cone-rod dystrophy and stargardt disease.. Mol Vis.

[5] Booij JC, van Soest S, Swagemakers SM, Essing AH, Verkerk AJ, van der Spek PJ, Gorgels TG, Bergen AA (2009). Functional annotation of the human retinal pigment epithelium transcriptome.. BMC Genomics.

[6] Howell GR, Macalinao DG, Sousa GL, Walden M, Soto I, Kneeland SC, Barbay JM, King BL, Marchant JK, Hibbs M, Stevens B, Barres BA, Clark AF, Libby RT, John SW (2011). Molecular clustering identifies complement and endothelin induction as early events in a mouse model of glaucoma.. J Clin Invest.

[7] Diehn JJ, Diehn M, Marmor MF, Brown PO (2005). Differential gene expression in anatomical compartments of the human eye.. Genome Biol.

[8] Schroeder A, Mueller O, Stocker S, Salowsky R, Leiber M, Gassmann M, Lightfoot S, Menzel W, Granzow M, Ragg T (2006). The RIN: An RNA integrity number for assigning integrity values to RNA measurements.. BMC Mol Biol.

[9] Miller CL, Diglisic S, Leister F, Webster M, Yolken RH (2004). Evaluating RNA status for RT-PCR in extracts of postmortem human brain tissue.. Biotechniques.

[10] Wang WH, McNatt LG, Shepard AR, Jacobson N, Nishimura DY, Stone EM, Sheffield VC, Clark AF (2001). Optimal procedure for extracting RNA from human ocular tissues and expression profiling of the congenital glaucoma gene FOXC1 using quantitative RT-PCR.. Mol Vis.

[11] Malik KJ, Chen CD, Olsen TW (2003). Stability of RNA from the retina and retinal pigment epithelium in a porcine model simulating human eye bank conditions.. Invest Ophthalmol Vis Sci.

[12] Wang JC, Wang A, Gao J, Cao S, Samad I, Zhang D, Ritland C, Cui JZ, Matsubara JA (2012). TECHNICAL BRIEF: Isolation of total DNA from postmortem human eye tissues and quality comparison between iris and retina.. Mol Vis.

